# Reduction of BDNF Levels and Biphasic Changes in Glutamate Release in the Prefrontal Cortex Correlate with Susceptibility to Chronic Stress-Induced Anhedonia

**DOI:** 10.1523/ENEURO.0406-23.2023

**Published:** 2023-11-20

**Authors:** Xiao Hu, Hui-Ling Zhao, Nurhumar Kurban, Yu Qin, Xi Chen, Su-Ying Cui, Yong-He Zhang

**Affiliations:** Department of Pharmacology, School of Basic Medical Science, Peking University, Beijing 100191, China

**Keywords:** anhedonia susceptibility, BDNF, chronic stress, glutamate release, ketamine, prefrontal cortex

## Abstract

Chronic stress has been considered to induce depressive symptoms, such as anhedonia, particularly in susceptible individuals. Synaptic plasticity in the prefrontal cortex (PFC) is closely associated with susceptibility or resilience to chronic stress-induced anhedonia. However, effects of chronic stress with different durations on the neurobiological mechanisms that underlie susceptibility to anhedonia remain unclear. The present study investigated effects of chronic mild stress (CMS) for 14, 21, and 35 d on anhedonia-like behavior and glutamate synapses in the PFC. We found that brain-derived neurotrophic factor (BDNF) levels in the PFC significantly decreased only in anhedonia-susceptible rats that were exposed to CMS for 14, 21, and 35 d. Additionally, 14 d of CMS increased prefrontal glutamate release, and 35 d of CMS decreased glutamate release, in addition to reducing synaptic proteins and spine density in the PFC. Moreover, we found that anhedonia-like behavior in a subset of rats spontaneously decreased, accompanied by the restoration of BDNF levels and glutamate release, on day 21 of CMS. Ketamine treatment restored the reduction of BDNF levels and biphasic changes in glutamate release that were induced by CMS. Our findings revealed a progressive reduction of synaptic plasticity and biphasic changes in glutamate release in the PFC during CMS. Reductions of BDNF levels may be key neurobiological markers of susceptibility to stress-induced anhedonia.

## Significance Statement

Chronic stress is generally recognized as a major risk factor for depressive disorders. Consistent with humans, only a subset of rodents develops depression-like phenotypes such as anhedonia when they are exposed to chronic stress. We investigated neurobiological effects at various stages of chronic stress to gain a deeper understanding of the mechanisms underlying stress-induced susceptibility to depression. Our findings indicate that reductions of brain-derived neurotrophic factor (BDNF) levels and biphasic changes in glutamate release in the prefrontal cortex (PFC) correlate with susceptibility to chronic stress-induced anhedonia. These findings reveal potential biomarkers of susceptibility to chronic stress and will contribute to the early prediction of depression and effective interventions.

## Introduction

Depression is a common mental illness that markedly impacts quality of life and imposes serious financial burdens on patients and society (World Health Organization, 2021). Chronic stress is generally recognized as a major risk factor for depressive disorders. However, responses to stress exhibit remarkable heterogeneity among individuals. Consistent with humans, only a subset of rodents develops depression-like phenotypes when they are subjected to stress procedures (Russo et al., 2012). Exploring the factors that are involved in susceptibility to stress will contribute to the early prediction of depression and effective interventions.

Proteomic studies revealed differences in presynaptic proteins that are involved in neurotransmitter release between chronic mild stress (CMS)-susceptible and CMS-resilient animals (Bisgaard et al., 2007; Henningsen et al., 2012). *VGluT1* knock-down, which leads to an increase in glutamate levels in the cortex, increases susceptibility to CMS in mice (Garcia-Garcia et al., 2009). Conversely, brain-derived neurotrophic factor (BDNF) hyperexpression increases resilience to CMS in rats (Taliaz et al., 2011). Furthermore, environmental enrichment promotes resilience by increasing dendritic branching and synaptogenesis in the cortex (van Praag et al., 2000). These results indicate the involvement of neurotrophic factors, synaptic plasticity, and endogenous excitatory amino acids in mediating susceptibility to stress. However, the neurobiological processes that underlie mood regulation are intricately connected and differ at various stages of chronic stress (Flores-Kanter et al., 2021).

BDNF is a neurotrophin that is critical for synapse formation in brain circuits that are involved in mood function. Chronic stress is associated with low BDNF expression and damage to synaptic plasticity, including dendritic atrophy, a reduction of synapse number, and volumetric changes in the prefrontal cortex (PFC; Duman and Monteggia, 2006; Phillips, 2017). Furthermore, abnormal glutamate transmission is closely related to the lower expression and release of BDNF (Murrough et al., 2017). However, the precise nature of stress-induced changes in glutamate transmission is not entirely clear. For example, acute stress rapidly enhances glutamate release and transmission (Bagley and Moghaddam, 1997; Treccani et al., 2014). Extracellular glutamate accumulation and deficits in synaptic proteins are detected after chronic unpredictable stress (Li et al., 2018). In contrast, certain chronic stress procedures reduce glutamate release and the frequency of spontaneous EPSCs in the PFC (Chiba et al., 2012; Son et al., 2018). We speculate that these inconsistent results may be related to differences in the duration of stress. However, few studies have focused on dynamic consequences of chronic stress. Accordingly, we investigated neurobiological effects at various stages of chronic stress to gain a deeper understanding of the mechanisms that underlie stress-induced susceptibility to depression.

Chronic mild stress is a commonly used rodent model of stress-induced anhedonia (Willner, 2016). Notably, the CMS model offers the flexibility of selecting various endpoints during the stress procedure, allowing investigations of the ways in which neurobiological processes that are associated with susceptibility to chronic stress develop over time.

Our study investigated effects of CMS with varying durations on synapses in the PFC. Fourteen, 21, and 35 d of CMS decreased BDNF levels in anhedonia-susceptible but not anhedonia-resilient rats. Deficits in synaptic structure were observed after 35 d but not 14 or 21 d of CMS. Moreover, in anhedonia-susceptible rats, glutamate release was enhanced after 14 d of CMS but was reduced after 35 d of CMS. These findings indicate that a reduction of BDNF levels is a common change in the PFC in CMS-susceptible rats, whereas the impact on synaptic structure and glutamate release appears to be influenced by the duration of CMS. Furthermore, we observed restorations of BDNF levels and glutamate release in rats whose anhedonia-like behavior spontaneously recovered. Ketamine treatment alleviated anhedonia and reversed CMS-induced changes in BDNF levels and glutamate release. These findings suggest a correlation between susceptibility to CMS-induced anhedonia and changes in BDNF levels, synaptic plasticity, and glutamate release in the PFC, offering potential strategies for the early prediction of stress-induced depression.

## Materials and Methods

### Animals

Male Wistar rats, aged six weeks and weighing between 180 and 200 g, were obtained from the Animal Center of Peking University in Beijing, China. The rats were housed in pairs, with two rats per cage, under standard laboratory conditions. The housing room maintained a temperature of 22–24°C, and the rats were exposed to a 12/12 h light/dark cycle (lights on from 9 A.M. to 9 P.M.). Throughout the study, the rats have free access to food and water, except during specific periods of exposure to CMS stressors. All experiments were in accordance with the National Research Council’s Guide for the Care and Use of Laboratory Animals and approved by the Peking University Animal Care and Use Committee (permission no. LA2020279).

### Drugs and treatments

Ketamine was obtained from Sigma-Aldrich. Ketamine (10 mg/kg) was dissolved in saline and was intraperitoneally injected at a constant volume of 5 ml/kg body weight.

### Chronic mild stress procedure

CMS was conducted with a variable sequence of different stressors that consisted of a random combination of two stressors per day. The stress procedure was slightly modified from previous studies (Willner et al., 1987), including food deprivation, water deprivation, lights off daytime, lights on overnight, 45° tilted cages, crowded housing, damp bedding, stroboscopic light and white noise. Food deprivation and water deprivation lasted 24 h, and the other stressors lasted 12 h. Rats were exposed to the CMS procedure for 14, 21, or 35 d.

### Sucrose preference test

Before conducting the main experiment, the baseline preference for sucrose solution of rats was examined. The adaptation phase involved a 24-h period during which the rats had access to two bottles, one filled with a 1% sucrose solution and the other with water. The position of the bottles was changed after 12 h to prevent any potential position preference. The rats were subjected to a 24-h period of food and water deprivation before the sucrose preference test (SPT). Following the deprivation period, the rats were given 1 h of free access to the two bottles, one containing 1% sucrose solution and the other containing water. The volume of consumed sucrose solution and water was meticulously recorded during this test. Sucrose preference was determined by calculating the ratio of the volume of sucrose solution consumed to the total volume of both sucrose solution and water consumed. Since all control rats exhibited a sucrose preference above 70%, preferences for sucrose below or above 70% were used as the criterion for categorizing rats as susceptible or resilient to CMS-induced anhedonia.

### Western blot analysis

After decapitating the rats, their PFC was rapidly removed. Tissues were homogenized in RIPA buffer with protease and phosphatase inhibitors, and were centrifuged at 12,000 × *g* for 15 min at 4°C. Supernatants were collected and their protein concentration was quantified using a BCA assay kit (Pierce). Equal amounts of protein were separated by 10% SDS-PAGE and transferred to polyvinylidene difluoride membranes (Millipore). The membranes were blocked with 5% BSA buffer for 1 h at room temperature, and then incubated with primary antibodies against BDNF (1:500; Abcam; rabbit), PSD-95 (1:1000; Cell Signaling; rabbit), synapsin-1 (1:1000; Cell Signaling; rabbit), and β-actin (1:10,000; ABclonal; rabbit) in TBST buffer (Tris-buffered saline + 0.1% Tween 20) at 4°C overnight. The membranes were then washed three times in TBST and incubated in peroxidase-labeled anti-rabbit (1:4000; ABclonal; goat) secondary antibody at room temperature for 2 h. The blots were then treated with an enhanced chemiluminescence detection kit (ABclonal). Western blot bands were scanned with a GelDoc XR System (Bio-Rad) and analyzed densitometrically using ImageJ. The results were normalized to the protein expression level of β-actin in each sample. Adjustments of brightness, contrast and image rotation are applied for clarity.

### Golgi staining

After decapitating the rats, their brains were rapidly removed. Golgi staining was conducted using the FD Rapid GolgiStain kit (FD Neuro Technologies) following the manufacturer’s instruction. Secondary apical dendrites of pyramidal neurons from the PFC region were captured using a 100× objective with a TCS SP8 confocal microscope (Leica Microsystems) as previously reported with slight modifications (Yang et al., 2015). For spine density measurements, all clearly evaluable areas containing 50–100 µm of secondary dendrites from each imaged neuron were used. Spines that emerged perpendicular to the dendritic shaft were counted and the length of the dendritic segment was measured to calculate spine density per 10 μm.

### Preparation of synaptosomes

After decapitating the rats, their PFC was rapidly removed. Purified synaptosomes were prepared as previously reported with slight modifications (Dunkley et al., 2008). The PFC tissue was homogenized using a glass Teflon tissue grinder (clearance 0.25 mm) in seven volumes of cold sucrose buffer (0.32 m sucrose, 1 mm EDTA, 0.25 mm dithiothreitol, pH 7.4) supplemented with protease and phosphatase inhibitors. The homogenate was centrifuged at 1000 × *g* for 5 min at 4°C to remove nuclei and cell debris. The supernatant was collected and loaded on top of discontinuous Percoll gradients. From top to bottom, the Percoll gradient comprised 2 ml each of 3%, 10%, 15%, and 23% Percoll v/v in Tris-buffered sucrose. The gradients were centrifuged at 33,500 × *g* for 5 min at 4°C. The layer between 15% and 23% Percoll (synaptosomal fraction) was carefully collected and washed to remove Percoll by centrifugation at 15,000 × *g* for 15 min in cold 0.32 m sucrose. The pellet was resuspended in physiological medium (128 mm NaCl, 3 mm KCl, 1.2 mm MgSO_4_, 1.2 mm CaCl_2_, 1.2 mm NaH_2_PO_4_, 5 mm NaHCO_3_, 10 mm HEPES, and 10 mm glucose, pH 7.4). Synaptosomal protein concentrations were determined using the Bradford assay.

### Glutamate release assay

The determination of endogenous glutamate release in synaptosomes was conducted as previously reported with slight modifications (Bonanno et al., 2005). The synaptosomal suspension (∼100 μg of protein) was layered on microporous filters at the bottom of a set of parallel chambers in a superfusion system with 95% O_2_ and 5% CO_2_ at 37°C and superfused at 0.5 ml/min with physiological medium for 36 min. When studying the depolarization-evoked release of glutamate, synaptosomes were exposed to 15 mm KCl for 120 s at *t* = 39 min. Samples were collected according to the following scheme: two 3-min samples (*t* = 36–39 min and *t* = 45–48 min; basal release) before and after one 6-min sample (*t* = 39–45 min; KCl-evoked release). The depolarization-evoked overflow of glutamate was estimated by subtracting the glutamate content of the two basal release samples from the KCl-evoked release sample.

Levels of glutamate were detected by reverse‐phase high‐performance liquid chromatography (HPLC; Dionex UltiMate 3000, Thermo Fisher Scientific) combined with precolumn derivatization with O‐phthalaldehyde (OPA) and fluorometric detection. Glutamate was derivatized with OPA prepared by dissolving 27 mg OPA in 9 ml of 0.1 m sodium tetraborate and 1 ml of 100% methanol to which 5 μl of β-mercaptoethanol was added. This solution was then diluted 1:3 with sodium tetraborate and 10 μl added to 20 μl of dialysate sample. The sample and derivatization solution reacted for 1 min before being injected onto a C18 column (75 × 3.0 mm, Shiseido). The amount of glutamate levels in the samples was expressed as picomoles per milligram of protein.

### Statistical analysis

The data were expressed as the mean ± SEM and were analyzed using SPSS 26.0 software (SPSS Inc.). The data were analyzed using one-way ANOVA followed by Tukey’s *post hoc* test. Probability values <0.05 (*p *<* *0.05) were considered statistically significant.

## Results

### Anhedonia-like behaviors induced by 14, 21, and 35 d of CMS showed U-shaped development

**Figure 1.**
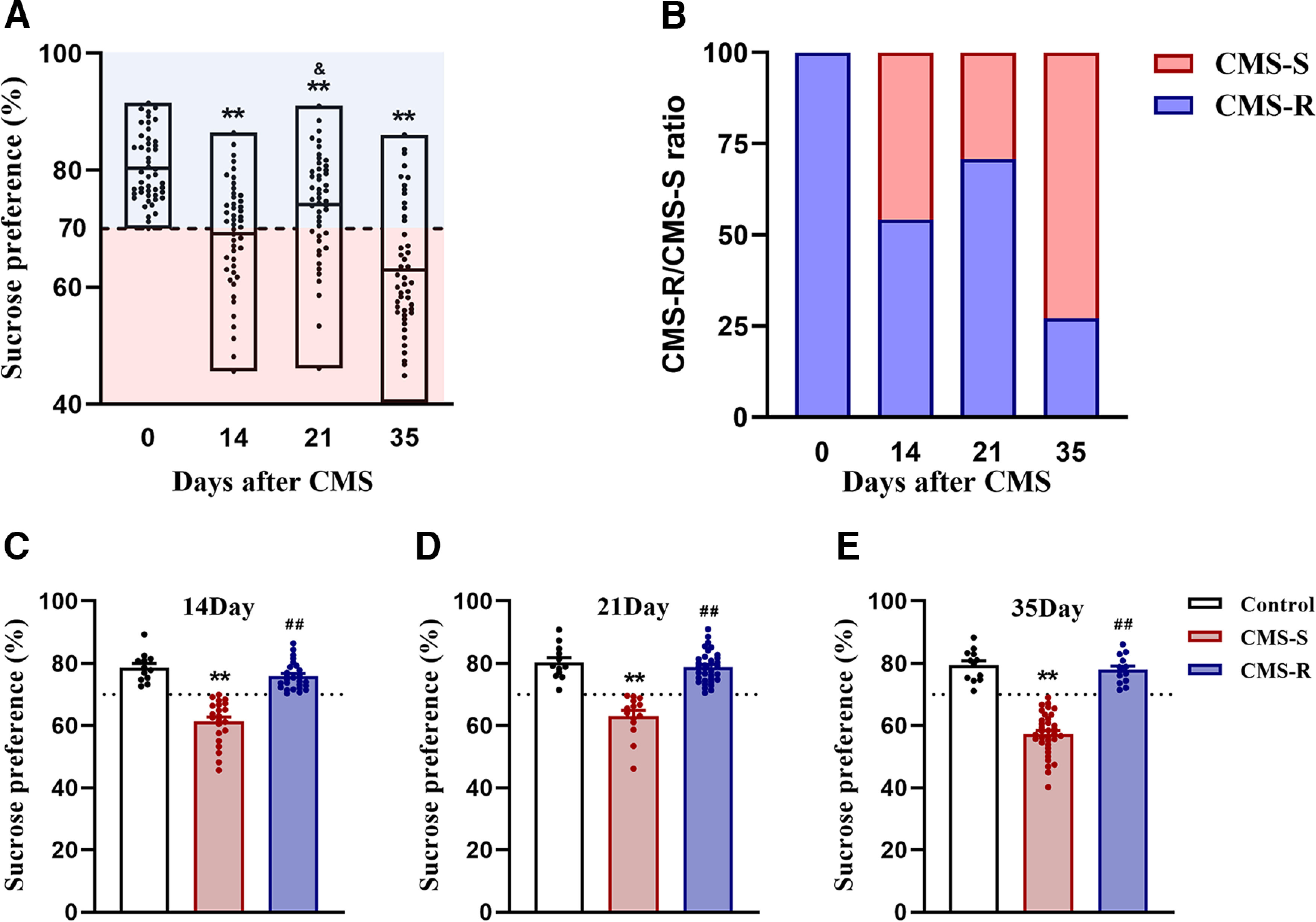
The effects of CMS on anhedonia-like behavior. Rats were exposed to CMS for 14, 21, and 35 d (*n* = 48/group) or handled as control (*n* = 12). Sucrose preference test was conducted 1 d after the end of each stress procedure. ***A***, Time course of changes in sucrose preference after 14, 21, or 35 d of CMS. Rats exposed to CMS were classified into two phenotypes: susceptible (CMS-S) and resilient (CMS-R) to anhedonia, based on a criterion of 70% preference for sucrose. ***B***, The proportion of anhedonia-susceptible rats after 14, 21, or 35 d of CMS. ***C–E***, Sucrose preference in rats after 14 d (*n* = CMS-S 22; CMS-R 26), 21 d (*n* = CMS-S 14; CMS-R 34), or 35 d of CMS (*n* = CMS-S 35; CMS-R 13). All data are presented as the mean ± SEM; **p <* *0.01 compared with control group, &*p *<* *0.05 compared with 14 d CMS group and ##*p *<* *0.01 compared with CMS-S group; one-way ANOVA followed by Tukey’s *post hoc* test.

To examine effects of CMS on anhedonia-like behavior, we conducted the SPT 1 d after completing the stress procedure. Fourteen, 21, and 35 d of CMS reduced sucrose preference (*F*_(3,188)_ = 32.506, *p *<* *0.001; Fig. 1*A*). Interestingly, rats that were exposed to CMS for 21 d exhibited higher sucrose preference than rats that were exposed to CMS for 14 d (Fig. 1*A*).

Considerable evidence suggests that, because of individual differences, only a proportion of rats are susceptible to CMS, whereas others are resilient (Willner, 2016). Rats with a sucrose preference lower than 70% were considered susceptible to CMS-induced anhedonia (CMS-S), whereas others were considered resilient (CMS-R). This criterion was based on the fact that none of the control rats exhibited ≤ 70% preference for sucrose. According to the criterion, 45.8% (22 of 48), 29.2% (14 of 48), and 72.9% (35 of 48) of the rats exhibited the anhedonia-susceptible phenotype following 14, 21, and 35 d of CMS, respectively (Fig. 1*B*). Fourteen, 21, and 35 d of CMS reduced sucrose preference only in the CMS-S group (14 d: *F*_(2,57)_ = 56.037, *p *<* *0.001; 21 d: *F*_(2,57)_ = 45.968, *p *<* *0.001; 35 d: *F*_(2,57)_ = 90.946, *p *<* *0.001; Fig. 1*C–E*). Our results indicate that the proportion of rats that exhibited the anhedonia-susceptible phenotype developed in a U-shaped pattern over time during the CMS procedure.

### Fourteen, 21, and 35 d of CMS decreased BDNF levels and caused progressive deficits in synaptic plasticity in the PFC in anhedonia-susceptible rats

**Figure 2.**
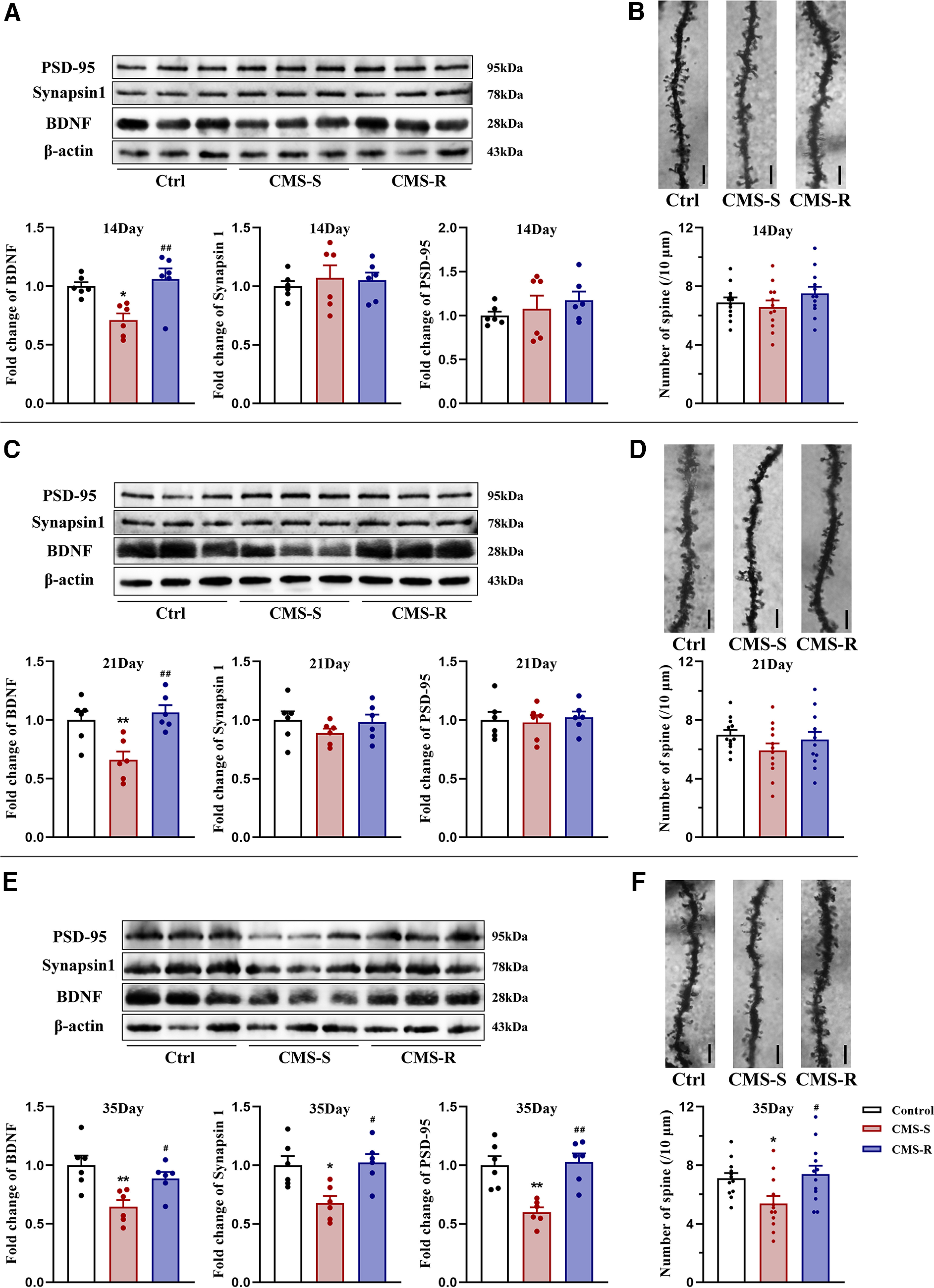
The effects of CMS on the levels of BDNF, synapsin-1, and PSD-95, as well as spine number in the PFC. Rats exposed to CMS for 14, 21, or 35 d were divided into CMS-S and CMS-R groups based on their sucrose preference. ***A***, ***C***, ***E***, Quantification of protein levels and representative Western blot images of BDNF, synapsin-1, and PSD-95 in the PFC in control, CMS-S, and CMS-R rats after14, 21, or 35 d of CMS. The results were normalized to the level of β-actin in each sample (*n* = 6/group). ***B***, ***D***, ***F***, Representative photomicrographs for Golgi staining and the number of spines in the PFC in control, CMS-S, and CMS-R rats after 14, 21, or 35 d of CMS (*n* = 3 neurons/rat, 4 rats/group). Scale bar = 10 μm. All data are presented as the mean ± SEM; *p <* *0.05, **p <* *0.01 compared with control group and #*p *<* *0.05, ##*p *<* *0.01 compared with CMS-S group; one-way ANOVA followed by Tukey’s *post hoc* test.

Chronic stress induces depression by disrupting synaptic plasticity in brain circuits that are involved in mood regulation. BDNF is an essential regulator of synaptic transmission and plasticity (Phillips, 2017). To investigate effects of CMS on BDNF expression, we examined levels of BDNF in the PFC by Western blotting. BDNF levels did not differ between control and anhedonia-resilient rats but significantly decreased in anhedonia-susceptible rats that were exposed to CMS for 14, 21, and 35 d (14 d: *F*_(2,15)_ = 8.378, *p *=* *0.004; 21 d: *F*_(2,15)_ = 9.655, *p *=* *0.002; 35 d: *F*_(2,15)_ = 7.692, *p *=* *0.005; Fig. 2*A*,*C*,*E*).

Chronic stress has been shown to have detrimental effects on synaptic plasticity (Parekh et al., 2022). Synapsin-1 and PSD-95 are essential synaptic proteins. Notably, levels of synapsin-1 and PSD-95 significantly decreased only in anhedonia-susceptible rats that were exposed to CMS for 35 d but not in any other rats that were exposed to CMS for 14 or 21 d (14 d: synapsin-1: *F*_(2,15)_ = 0.219, *p *=* *0.806; PSD-95: *F*_(2,15)_ = 0.654, *p *=* *0.534. 21 d: synapsin-1: *F*_(2,15)_ = 0.967, *p *=* *0.403; PSD-95: *F*_(2,15)_ = 0.124, *p *= 0.884. 35 d: synapsin-1: *F*_(2,15)_ = 7.301, *p *=* *0.006; PSD-95: *F*_(2,15)_ = 13.23, *p *<* *0.001; Fig. 2*A*,*C*,*E*).

We further examined synaptic spine density in the PFC by Golgi staining. Consistent with the results of synapse-associated proteins, prefrontal spine density was reduced only in anhedonia-susceptible rats that were exposed to CMS for 35 d, whereas no significant changes were observed in rats that were exposed to CMS for 14 or 21 d (14 d: *F*_(2,33)_ = 1.303, *p *=* *0.285; 21 d: *F*_(2,33)_ = 1.459, *p *=* *0.247; 35 d: *F*_(2,33)_ = 4.857, *p *=* *0.014; Fig. 2*B*,*D*,*F*).

Altogether, our results indicate that CMS-induced impairments in synaptic plasticity in the PFC occurred specifically in anhedonia-susceptible rats that were exposed to long-term stress. These deficits in synaptic plasticity exhibited a progressive characteristic, with a reduction of BDNF levels that preceded synaptic atrophy during the stress procedure.

### Fourteen and 35 d of CMS induced biphasic changes in glutamate release in the PFC in anhedonia-susceptible rats

**Figure 3.**
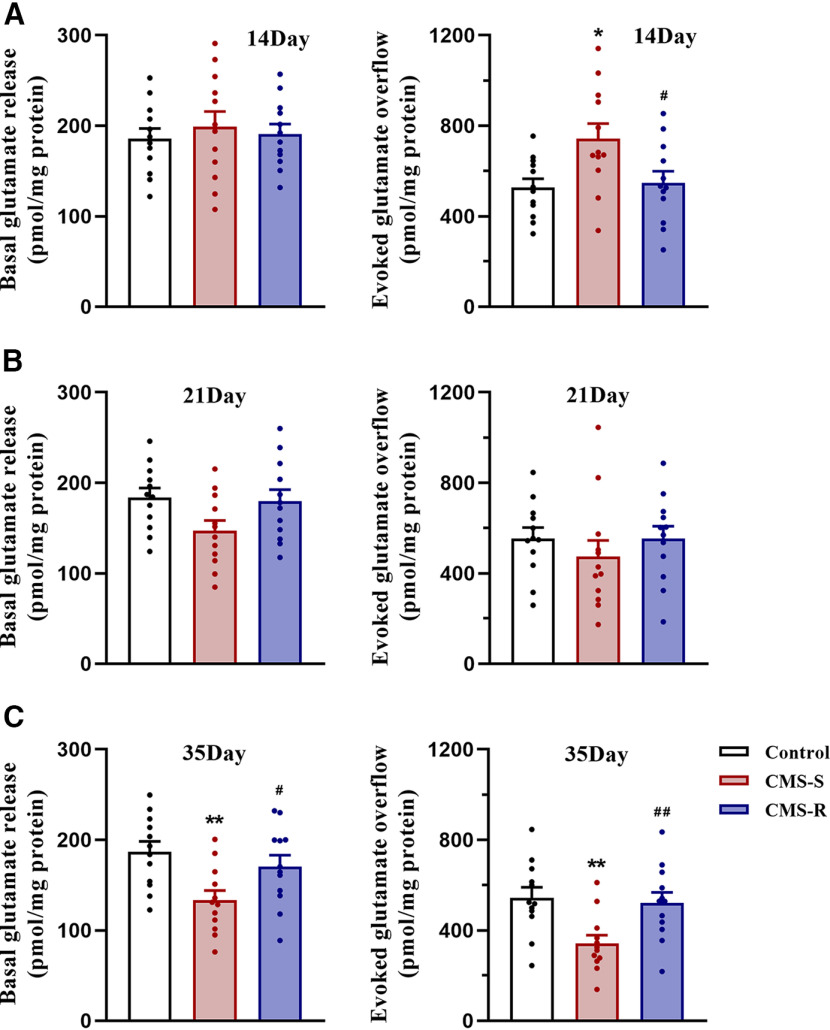
The effects of CMS on the basal and evoked glutamate release in the PFC. Rats exposed to CMS for 14, 21, or 35 d were divided into CMS-S and CMS-R groups based on their preference for sucrose. ***A–C***, The basal and depolarization-evoked glutamate release in the PFC in control, CMS-S, and CMS-R rats after 14, 21, or 35 d of CMS (*n* = 12/group). All data are presented as the mean ± SEM; *p <* *0.05, **p <* *0.01 compared with control group and #*p *<* *0.05, ##*p *<* *0.01 compared with CMS-S group; one-way ANOVA followed by Tukey’s *post hoc* test.

Chronic stress-induced changes in glutamate synapses are considered to play an important role in the development of depression (Sanacora et al., 2022). Synaptosomes are suitable for examining effects of chronic stress on synaptic glutamate release because they contain complete presynaptic terminals (Dunkley et al., 2008). To investigate effects of CMS on the presynaptic release of endogenous glutamate, we isolated purified synaptosomes from the PFC and determined basal and depolarization-evoked (induced by 15 mm KCl) glutamate release in synaptosomes by superfusion. This method allows determination of the exocytotic release of glutamate while preventing reuptake by glutamate transporters or the activation of synaptic receptors (Musazzi et al., 2015). Both basal and depolarization-evoked glutamate release did not differ between control and anhedonia-resilient rats that were exposed to CMS for 14, 21, or 35 d (Fig. 3*A–C*). Basal glutamate release did not change in rats that were exposed to CMS for 14 or 21 d (Fig. 3*A*,*B*), but it decreased in anhedonia-susceptible rats that were exposed to CMS for 35 d (14 d: *F*_(2,33)_ = 0.244, *p *=* *0.785; 21 d: *F*_(2,33)_ = 3.041, *p *=* *0.061; 35 d: *F*_(2,33)_ = 5.501, *p *=* *0.009; Fig. 3*C*). Interestingly, 14 d of CMS enhanced depolarization-evoked glutamate release in anhedonia-susceptible rats (*F*_(2,33)_ = 4.967, *p *=* *0.013; Fig. 3*A*). In contrast, 21 d of CMS did not induce significant changes in depolarization-evoked glutamate release (*F*_(2,33)_ = 0.604, *p *=* *0.553; Fig. 3*B*), but 35 d of CMS reduced depolarization-evoked glutamate release in anhedonia-susceptible rats (*F*_(2,33)_ = 6.336, *p *= 0.005; Fig. 3*C*). Our results indicate that CMS induces changes in glutamate release in the PFC only in anhedonia-susceptible rats. Changes in glutamate release exhibit a biphasic pattern over time. Prefrontal glutamate release is enhanced in the early stage of CMS, but it becomes reduced in a later stage.

### BDNF levels and glutamate release in the PFC returned to normal in rats whose anhedonia-like behavior was spontaneously relieved on day 21 of CMS

**Figure 4.**
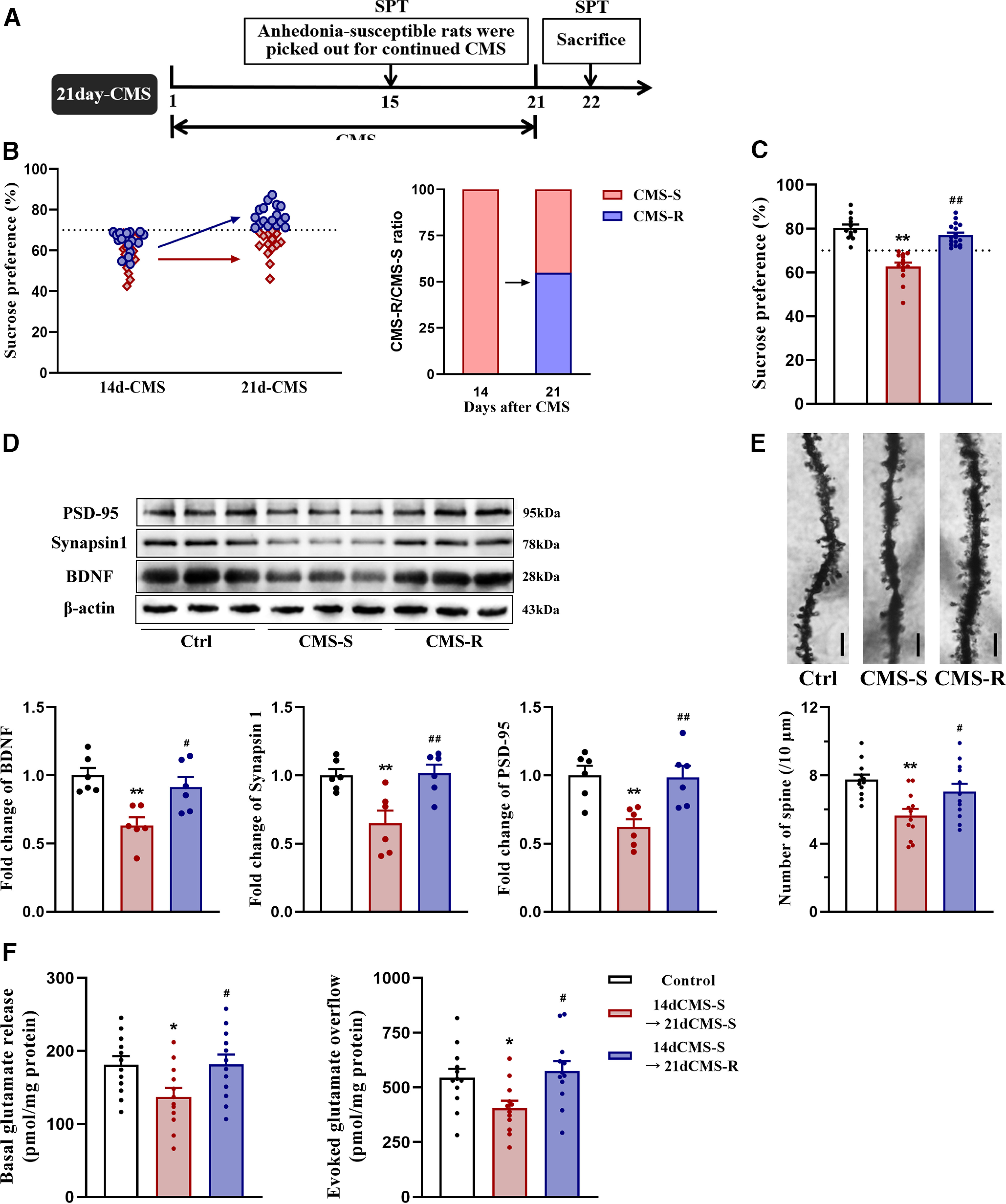
The effects of prolonged CMS on anhedonia-like behavior, levels of BDNF and synaptic proteins, spine density, and glutamate release in the PFC in former anhedonia-susceptible rats. ***A***, Schedule of experiment. Rats were exposed to CMS for 21 d, and those susceptible to anhedonia were picked out by SPT on day 15. A second round of SPT was conducted on day 22 to reexamine their susceptibility to anhedonia. Rats were re-divided into CMS-S and CMS-R groups based on a 70% preference for sucrose as the criterion. ***B***, The proportion of anhedonia-susceptible rats after 21 d of CMS. ***C***, Sucrose preference in rats after 21 d of CMS (*n* = control 12; CMS-S 14; CMS-R 17). ***D***, Quantification of protein levels and representative Western blot images of BDNF, synapsin-1, and PSD-95 in the PFC in rats after 21 d of CMS. The results were normalized to the level of β-actin in each sample (*n* = 6/group). ***E***, Representative photomicrographs for Golgi staining and the number of spines in the PFC in rats after 21 d of CMS (*n* = 3 neurons/rat, 4 rats/group). Scale bar = 10 μm. ***F***, The basal and depolarization-evoked glutamate release in the PFC in rats after 21 d of CMS (*n* = 12/group). All data are presented as the mean ± SEM; *p <* *0.05, **p <* *0.01 compared with control group and #*p *<* *0.05, ##*p *<* *0.01 compared with CMS-S group; one-way ANOVA followed by Tukey’s *post hoc* test.

Adaptive recovery from chronic stress is crucial for maintaining healthy affective functioning (Flores-Kanter et al., 2021). Our previous results showed a reduction of the proportion of the anhedonia-susceptible phenotype in rats that were exposed to CMS for 21 d compared with rats that were exposed to CMS for 14 d (Fig. 1*B*). Based on these results, we hypothesized that anhedonia-like behavior in a subset of susceptible rats could be relieved at some time point during the CMS procedure. To test this hypothesis, we chose rats that exhibited anhedonia-like behavior after 14 d of CMS by the SPT. These rats continued to be exposed to CMS until day 21, and their susceptibility to anhedonia was examined again by the SPT (Fig. 4*A*). Among these rats, 54.8% (17 of 31) exhibited an attenuation of anhedonia-like behavior, but others still exhibited anhedonia-like behavior (*F*_(2,40)_ = 37.348, *p *<* *0.001; Fig. 4*B*,*C*). Our results provide evidence of an adaptation to stress that contributes to the restoration of hedonic capacity during a certain period of CMS. These findings also suggest the presence of neurobiological processes that enable certain individuals to recover from the impact of chronic stress.

Potential correlations were found between anhedonia and changes in BDNF levels and glutamate release in the PFC (Figs. 2, 3). To further investigate the neurobiological process that underlies susceptibility and resilience to anhedonia, we examined levels of BDNF, synapsin-1, and PSD-95, spine density, and basal and depolarization-evoked glutamate release in the PFC in rats whose anhedonia-like behavior spontaneously decreased on day 21 of CMS. Levels of BDNF, synapsin-1, and PSD-95 (BDNF: *F*_(2,15)_ = 9.245, *p *=* *0.002; synapsin-1: *F*_(2,15)_ = 8.96, *p *=* *0.003; PSD-95: *F*_(2,15)_ = 8.852, *p *=* *0.003; Fig. 4*D*), spine density (*F*_(2,33)_ = 7.536, *p *=* *0.002; Fig. 4*E*), as well as basal and depolarization-evoked glutamate release decreased in rats that remained susceptible to anhedonia (basal: *F*_(2,33)_ = 4.179, *p *=* *0.024; evoked: *F*_(2,33)_ = 5.145, *p *=* *0.011; Fig. 4*F*). Instead, no significant changes were observed between control rats and rats that became resilient to anhedonia. Our results indicate that the restoration of BDNF levels, synaptic plasticity, and glutamate release in the PFC may be signs of successful adaptation and the consequent relief of anhedonia. Conversely, persistence changes in BDNF levels, synaptic plasticity, and glutamate release in the PFC may indicate a failure of adaptation to stress and correlate with a persistent state of anhedonia.

### Ketamine treatment restored the decrease in BDNF levels and biphasic changes in glutamate release in the PFC in anhedonia-susceptible rats

**Figure 5.**
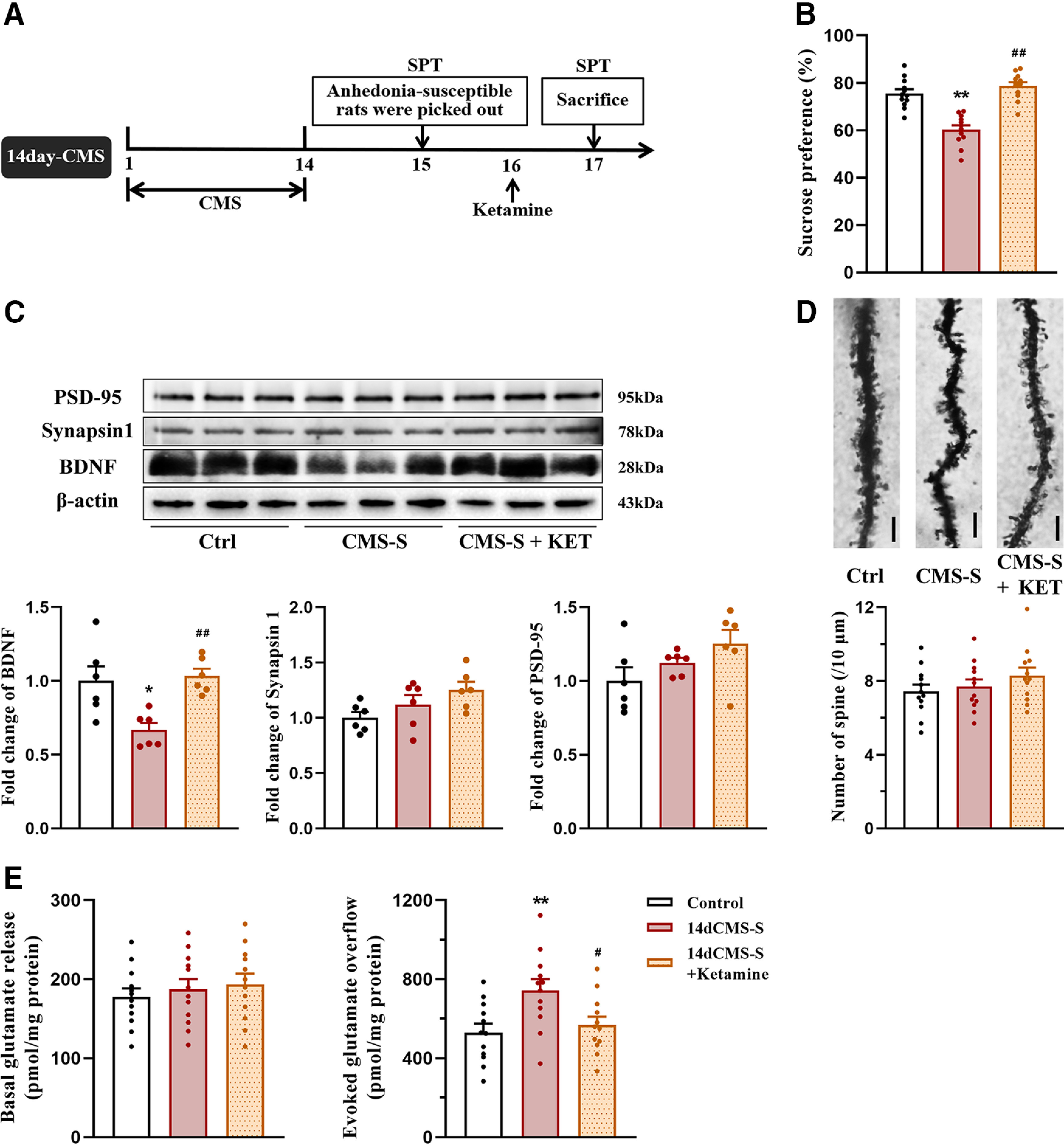
The effects of ketamine on anhedonia-like behavior, levels of BDNF and synaptic proteins, spine density, and glutamate release in the PFC in rats after 14 d of CMS. ***A***, Schedule of experiment. Rats were exposed to CMS for 14 d, and those susceptible to anhedonia were picked out by SPT on day 15. The anhedonia-susceptible rats were injected with saline or ketamine (10 mg/kg) on day 16, and their anhedonia-like behaviors were examined by SPT on day 17. ***B***, Sucrose preference in rats after 14 d of CMS and ketamine treatment (*n* = 12/group). ***C***, Quantification of protein levels and representative western blot images of BDNF, synapsin-1, and PSD-95 in the PFC in rats after 14 d of CMS and ketamine treatment. The results were normalized to the level of β-actin in each sample (*n* = 6/group). ***D***, Representative photomicrographs for Golgi staining and the number of spines in the PFC in rats after 14 d of CMS and ketamine treatment (*n* = 3 neurons/rat, 4 rats/group). Scale bar = 10 μm. ***E***, The basal and depolarization-evoked glutamate release in the PFC in rats after 14 d of CMS and ketamine treatment (*n* = 12/group). All data are presented as the mean ± SEM; *p <* *0.05, **p <* *0.01 compared with control group and #*p *<* *0.05, ##*p *<* *0.01 compared with CMS-S + vehicle group; one-way ANOVA followed by Tukey’s *post hoc* test.

Glutamate synapses in the PFC are considered critical targets that underlie ketamine’s antidepressant effect. The neurobiological mechanism by which ketamine mediates rapid and sustained antidepressant actions involves the regulation of glutamate release and synaptic plasticity in the PFC (Pham and Gardier, 2019). Our previous results showed that 14 and 35 d of CMS decreased BDNF levels and biphasic changes in glutamate release in the PFC in anhedonia-susceptible rats (Figs. 2, 3). We further investigated whether treatment with ketamine reverses these changes. Rats that exhibited anhedonia-like behavior after 14 d of CMS were selected by the SPT, and half of them were treated with ketamine (Fig. 5*A*). Ketamine treatment restored sucrose preference that was reduced by 14 d of CMS (*F*_(2,33)_ = 31.878, *p *<* *0.001; Fig. 5*B*). Ketamine also restored the lower levels of BDNF (BDNF: *F*_(2,15)_ = 8.48, *p *=* *0.003; synapsin-1: *F*_(2,15)_ = 3.19, *p *= 0.07; PSD-95: *F*_(2,15)_ = 2.552, *p *=* *0.111; spine density: *F*_(2,15)_ = 1.213, *p *=* *0.31; Fig. 5*C*,*D*) and the enhancement of depolarization-evoked glutamate release that were induced by 14 d of CMS (basal: *F*_(2,33)_ = 0.417, *p *=* *0.622; evoked: *F*_(2,15)_ = 8.48, *p *=* *0.003; Fig. 5*E*).

**Figure 6.**
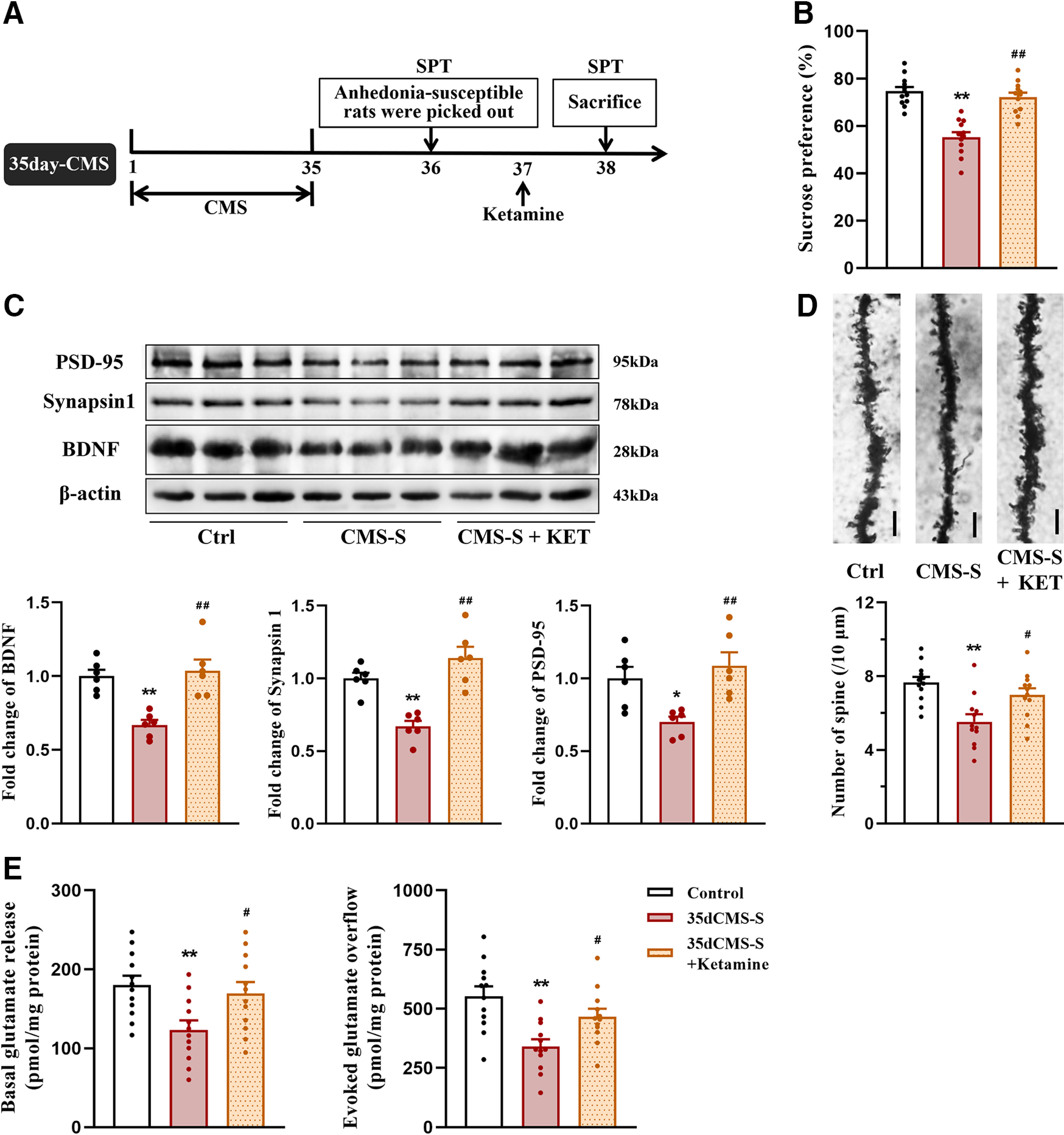
The effects of ketamine on anhedonia-like behavior, levels of BDNF and synaptic proteins, spine density, and glutamate release in the PFC in rats after 35 d of CMS. ***A***, Schedule of experiment. Rats were exposed to CMS for 35 d, and those susceptible to anhedonia were picked out by SPT on day 36. The anhedonia-susceptible rats were injected with saline or ketamine (10 mg/kg) on day 37, and their anhedonia-like behaviors were examined by SPT on day 38. ***B***, Sucrose preference in rats after 35 d of CMS and ketamine treatment (*n* = 12/group). ***C***, Quantification of protein levels and representative western blot images of BDNF, synapsin-1, and PSD-95 in the PFC in rats after 35 d of CMS and ketamine treatment. The results were normalized to the level of β-actin in each sample (*n* = 6/group). ***D***, Representative photomicrographs for Golgi staining and the number of spines in the PFC in rats after 35 d of CMS and ketamine treatment (*n* = 3 neurons/rat, 4 rats/group). Scale bar = 10 μm. ***E***, The basal and depolarization-evoked glutamate release in the PFC in rats after 35 d of CMS and ketamine treatment (*n* = 12/group) All data are presented as the mean ± SEM; *p <* *0.05, **p <* *0.01 compared with control group and #*p *<* *0.05, ##*p *<* *0.01 compared with CMS-S + vehicle group; one-way ANOVA followed by Tukey’s *post hoc* test.

Rats that exhibited anhedonia-like behavior after 35 d of CMS were selected by the SPT, and half of them were treated with ketamine (Fig. 6*A*). Ketamine restored sucrose preference that was reduced by 35 d of CMS (*F*_(2,33)_ = 29.233, *p *<* *0.001; Fig. 6*B*). Ketamine also restored the lower levels of BDNF, synapsin-1, and PSD-95 (BDNF: *F*_(2,15)_ = 14.148, *p *<* *0.001; synapsin-1: *F*_(2,15)_ = 19.636, *p *<* *0.001; PSD-95: *F*_(2,15)_ = 7.803, *p *= 0.005; Fig. 6*C*), the decreases in spine density (*F*_(2,33)_ = 9.198, *p *=* *0.001; Fig. 6*D*), and the reduction of basal and depolarization-evoked glutamate release that were induced by 35 d of CMS (basal: *F*_(2,33)_ = 5.742, *p *=* *0.007; evoked: *F*_(2,33)_ = 9.062, *p *=* *0.001; Fig. 6*E*). Altogether, these results indicate that the antidepressant effect of ketamine may correlate with the restoration of BDNF levels and normalization of glutamate release in the PFC. The remodeling of homeostatic transmission and plasticity in glutamate synapses may contribute to improvements in anhedonia-like behavior that is induced by chronic stress.

## Discussion

**Figure 7.**
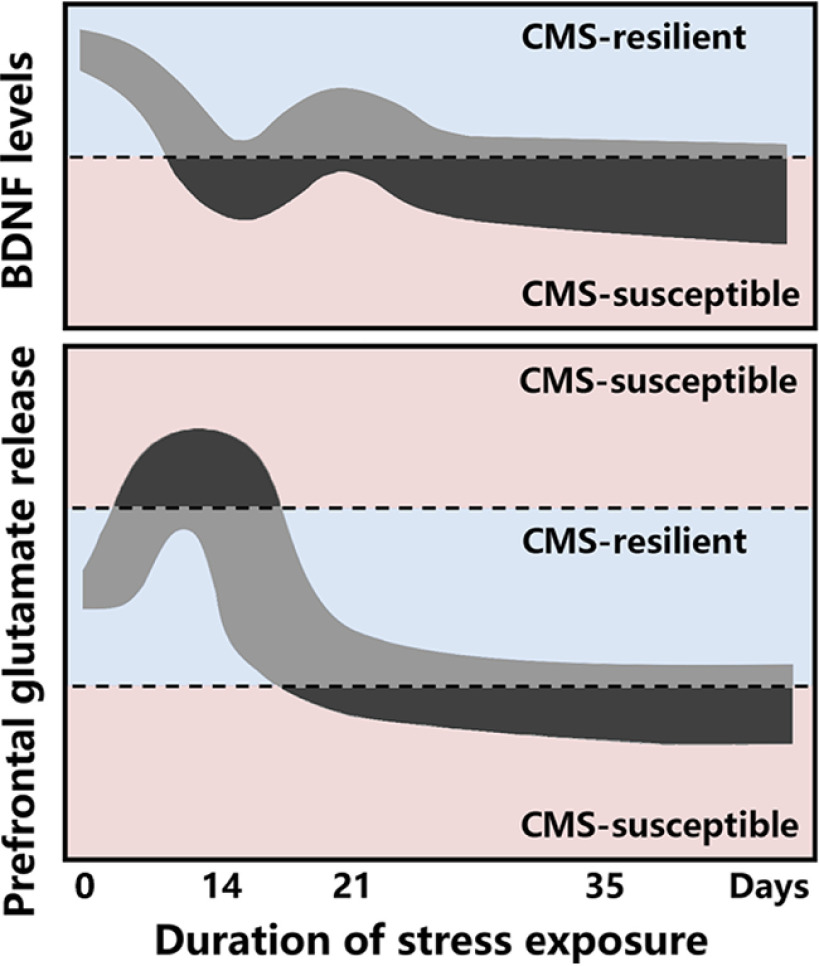
Time course of the effects of CMS on BDNF levels and glutamate release in the PFC. BDNF levels in the PFC are decreased in rats that are susceptible to CMS-induced anhedonia. Prefrontal glutamate release in CMS-susceptible rats is enhanced after 14 d of CMS, but it becomes reduced after 35 d of CMS. Anhedonia-like behavior in CMS-susceptible rats can spontaneously decrease, accompanied by restoration of BDNF levels and glutamate release, on day 21 of CMS. In contrast, BDNF levels and glutamate release in the PFC remain normal in rats that are resilient to CMS-induced anhedonia.

Converging evidence indicates that chronic stress leads to a series of structural and functional maladaptive changes that are associated with depression. Disturbances in the glutamatergic system and deficits in synaptic plasticity in the PFC have been found to play an important role in these processes (Parekh et al., 2022). Adaptive regulation during periods of confrontation with stressors is crucial for building resilience, whereas the persistence of maladaptive changes after stress often leads to depression (Flores-Kanter et al., 2021). Similar to humans, chronic stress leads to the development of depression-like behavior only in a subset of rodents (Willner, 2016). However, few studies have specifically explored differences in neurobiological responses between individuals that are susceptible or resilient to chronic stress. In the present study, we used 14, 21, and 35 d of CMS to distinguish anhedonia-susceptible and -resilient rats. We observed a U-shaped pattern in the development of the anhedonia-susceptible phenotype over the duration of CMS, indicating the potential spontaneous relief of anhedonia at a certain stage of CMS, such as on day 21. Indeed, >50% of rats exhibited the spontaneous remission of anhedonia-like behavior when CMS was extended from 14 to 21 d. Notably, the results showed that only anhedonia-susceptible rats exhibited decreases in BDNF levels, a progressive reduction of synaptic plasticity, and biphasic alterations of glutamate release in the PFC, suggesting that these changes may be markers of susceptibility to chronic stress (Fig. 7).

According to the neuroplasticity hypothesis of depression, stress induces the dysfunction of glutamate transmission and a reduction of neurotrophic factors, subsequently leading to morphologic changes in neurons, such as a shortening of dendrite length and a decrease in the number and density of spines (Duman and Monteggia, 2006). This hypothesis is supported by extensive studies that reported that stress and depression led to lower serum levels of BDNF and decreased the expression of BDNF in brain regions that are involved in the regulation of mood and cognition (Mousten et al., 2022; Cavaleri et al., 2023). BDNF is a vital signaling molecule in the brain that is responsible for synapse formation, synaptic plasticity, neurotransmission, and resistance to neuronal stress (Marosi and Mattson, 2014). There is a complex interaction between BDNF and glutamate transmission. Glutamate stimulates the expression and release of BDNF, which in turn modifies neuronal glutamate sensitivity, Ca^2+^ homeostasis, and plasticity. BDNF also directly modifies glutamate signaling by altering the expression of glutamate receptor subunits, including NMDA receptors (NMDARs) and AMPAR receptors (AMPARs; Murrough et al., 2017; Mattson, 2008). In the present study, we investigated changes in BDNF levels and glutamate release over the duration of CMS and assessed their correlation with depression-like behaviors. Throughout all stages of the CMS procedure (14, 21, and 35 d), only anhedonia-susceptible rats exhibited reductions of BDNF levels. Instead, anhedonia-resilient rats did not exhibit such changes. BDNF levels even returned to normal in rats whose anhedonia-like behavior was spontaneously relieved during CMS. These findings suggest that lower BDNF levels in the PFC are a persistent state that is associated with depression-like behavior. Consistent with BDNF levels, CMS-induced changes in glutamate release were only observed in rats that were susceptible to anhedonia and not in resilient rats. These findings suggest that abnormal BDNF levels and glutamate release in the PFC may correlate with susceptibility to stress-induced anhedonia. Notably, however, 14 d of CMS enhanced depolarization-evoked glutamate release, whereas 35 d of CMS reduced basal and depolarization-evoked glutamate release in anhedonia-susceptible rats. Although anhedonia occurred after both 14 and 35 d of CMS, we cautiously speculate that the neurobiological processes that underlie these two depression-like phenotypes are different.

In our previous study, we found that rats that were exposed to 7 and 21 d of corticosterone exhibited depression-like behavior, whereas those that were exposed to 14 d of corticosterone exhibited normal behavior, suggesting a U-shaped depressive effect that was mediated by corticosterone (Ding et al., 2018). This implies that chronic stress with different durations may lead to distinct behavioral and neurobiological effects. Generally, in the CMS paradigm, rats are exposed to at least four weeks of unpredictable mild stressors (Ramaker and Dulawa, 2017). We selected day 14 as one of the endpoints in the present study, representing an earlier time point than the standard paradigm. We found that depolarization-evoked glutamate release increased in the PFC in anhedonia-susceptible rats. This suggests that synaptic responses to chronic stress at an early stage manifest as an abnormal enhancement of glutamate release and excitatory transmission, which may lead to excitotoxicity-associated neuronal damage and consequently suppress the expression of BDNF (Zorumski and Olney, 1993; Autry et al., 2011). Notably, although these rats exhibited a reduction of BDNF levels, no significant changes in levels of synapsin-1 or PSD-95 or spine density were observed, implying that synaptic structures had not been damaged by stress. The intactness of synaptic structures also suggests that rats could spontaneously recover from the depression-like state. Interestingly, prefrontal glutamate release exhibited biphasic changes during CMS. When the CMS procedure was extended to 35 d, both basal and depolarization-evoked glutamate release decreased in the PFC in anhedonia-susceptible rats. In addition to the reduction of BDNF levels, chronic stress at a later stage also led to damage to synaptic structures in the form of lower levels of synapsin-1 and PSD-95 and spine density. The decrease in glutamate transmission directly downregulates the synthesis of synaptic proteins that is mediated by NMDARs and AMPARs, including BDNF, resulting in detrimental effects on plasticity and synaptic structures (Sanacora et al., 2022). As a consequence of the reduction of BDNF, impairments in synaptic plasticity would, in turn, further interfere with glutamate transmission, and this vicious cycle may be involved in the development of depression-like behavior. Moreover, rats whose anhedonia-like behavior was spontaneously relieved on day 21 of CMS appeared to temporarily break this vicious cycle and extricate themselves from the maladaptive state because BDNF levels, synaptic proteins, and glutamate release in the PFC returned to normal. Altogether, our study indicates that BDNF levels in the PFC are associated with maladaptive changes after stress that lead to depression and with adaptive regulation during the confrontation against stress to gain resilience. However, no significant changes in prefrontal glutamate release were observed in anhedonia-susceptible rats that were exposed to CMS for 21 d, suggesting that changes in glutamate release are more likely a sign of maladaptation to stress rather than a determining factor for stress-induced anhedonia.

Our study suggests that the potential self-regulatory mechanisms in the process of chronic stress may correlate with dynamic changes in BDNF levels and glutamate release in the PFC. The correlation between the reduction of BDNF levels and a depressive state supports the hypothesis that BDNF levels could serve as an indicator to predict the response to antidepressant treatment. Both traditional antidepressants, such as selective norepinephrine reuptake inhibitors and selective serotonin reuptake inhibitors, as well as rapid-acting antidepressants, such as ketamine, have been shown to increase BDNF levels in serum, the hippocampus, and the PFC (Duman and Monteggia, 2006; Castrén and Kojima, 2017; Kishi et al., 2018). Additionally, rapid and long-lasting antidepressant actions of ketamine are also closely related to improvements in glutamate transmission (Pham and Gardier, 2019). Consistent with previous studies, we found that a single administration of ketamine ameliorated anhedonia-like behavior that was induced by 14 and 35 d of CMS, accompanied by the restoration of BDNF levels in the PFC. Considering that BDNF levels in the PFC are decreased by stress and increased by antidepressants, BDNF may serve as a potential biomarker to assess the antidepressant treatment response. Moreover, we found that biphasic changes in glutamate release that were induced by CMS were restored following ketamine treatment, suggesting that ketamine induces the remodeling of glutamate transmission homeostasis rather than simply leading to an enhancement or a reduction of glutamate release. The simultaneous recovery from altered glutamate release and anhedonia-like behavior indicates a potential correlation.

In conclusion, our results indicate that chronic stress reduces BDNF levels and causes biphasic changes in glutamate release only in the PFC in anhedonia-susceptible rats. Furthermore, these changes return to normal in rats that exhibit the spontaneous relief of anhedonia-like behavior or receive ketamine treatment. Our study highlights a strong correlation between susceptibility to CMS-induced anhedonia and BDNF levels in the PFC, supporting the notion that BDNF could serve as a potential biomarker for assessing susceptibility to stress.

## Synthesis

Reviewing Editor: Sam Golden, The University of Washington Decisions are customarily a result of the Reviewing Editor and the peer reviewers coming together and discussing their recommendations until a consensus is reached. When revisions are invited, a fact-based synthesis statement explaining their decision and outlining what is needed to prepare a revision will be listed below. The following reviewer(s) agreed to reveal their identity: NONE. Note: If this manuscript was transferred from JNeurosci and a decision was made to accept the manuscript without peer review, a brief statement to this effect will instead be what is listed below.

Dear Authors,

Your manuscript was transferred to eNeuro from The Journal of Neuroscience after receiving comments from two qualified reviewers. They both found your work to be technically strong and with merit. Therefore, providing that an appropriate point-by-point rebuttal and updated manuscript is submitted, your article may be approved for publication following another round of editor-only review. Below please find the previous JN reviews, which must be fully and appropriately addressed.

Reviewer #1 Review

Rationale for Significance Rating for Authors (Required):

The manuscript entitled “Reduction of BDNF levels and biphasic changes in glutamate release in the prefrontal cortex correlate with susceptibility to chronic stress-induced anhedonia,” by Zhang et al. examines important physiological differences in animals that are either resilient or susceptible to stress. The main finding is that animals susceptible to stress display reduced BDNF levels and biphasic changes in glutamate release in the prefrontal cortex. The study is well designed and carefully carried out, however does not pose a significant advancement in the field and therefore would be better suited for another journal. Factors like BDNF levels, glutamatergic signaling, and the effect of antidepressants in susceptibility to stress have been extensively studied (DOI: 10.1016/j.biopsych.2014.01.013 ; DOI: 10.1016/j.pnpbp.2019.109682). Zhang et al. do not provide any mechanistic insight to their correlational observations.

Assessment of the Manuscript for Authors (Required):

Minor concerns:

1. Behavioral studies were performed for maximum of 35 days, which does not seem like a long enough timeline to be considered a longitudinal study. All references to “longitudinal” should be removed throughout the paper.

2. Figure 1A should show each data point, so that individual differences between subjects can be appreciated by the reader (like it was done in Figure 4B).

3. It is unclear why the ∼28 kDa BDNF band was quantified in Western blots. Could the authors also include the ∼14 kDa band, corresponding to the mature protein.

4. Figures 2-6, unclear which statistical test was utilized and should be specified in the figure legends.

5. Line 236, “Fourteen” should be “14.”

Reviewer #2 Review

Rationale for Significance Rating for Authors (Required):

Longitudinal analysis of CMS, i.e., characterization at three different time points after start of CMS. Rescue of susceptible phenotype by ketamine.

Assessment of the Manuscript for Authors (Required):

Review JN paper

The study by Hu and colleagues investigated the effects of chronic mild stress (CMS) after three different durations, i.e., after 14, 21, and 35 days, regarding one behavioural outcome (anhedonia-like behavior), the levels of BDNF (analyzed by Western blot) and glutamate release (using synaptosomes preparations). Based on the behavioral outcome, the authors stratified the animals into a resilient and a susceptible group. The general experimental design contains an interesting aspect, as commonly the duration of stress has not been considered much as a variable. Interestingly, dynamic features of the measured parameters were observed. In order to influence the outcome and concomitant BDNF levels and glutamate release, ketamine was applied to alleviate anhedonic-like behavior. In fact, restoration of BDNF levels and glutamate release was observed.

Major points

1) The behavioural outcome is fixed on one single behavior. Why not including e.g., forced swim test, tail suspension test, and anxiety tests (open field)?

2) Please give the details and reasoning of the stratification into resilient and susceptible animals in the method part.

3) Figure 2B,D,F: Which subpart of PFC was analysed: prelimbic or infralimbic cortex? These two regions contain different functions.

4) In the context of BDNF and resilience, it would have been very interesting to investigate the levels of BDNF in Nucleus accumbens and hippocampus. Are there data on those brain areas?

5) What are the expression levels of pro-BDNF?

6) Figures 5 and Figure 6: What was the reasoning not to wait more days after ketamine treatment, e.g., 3-4 days in order to see longer-term effects. Is there an interference of food/water deprivation and ketamine treatment?

7) Are there data on the morphology in the PFC (spine density) upon ketamine? This would be an additional indication that ketamine hooks up the pathways investigated in the previous part of the study. (see point 6 above)

8) Figure 7: The levels before Day 14 presumably represent the basal state. Please indicate x axis the Zero point. It would be helpful to integrate the ketamine experiment into the figure.

Minor points

1) Abstract / line 39: rather “elucidate a mechanism”. However, characterizing a biomarker does not necessarily equal with a mechanism. If BDNF is seen as a biomarker (as measured in isolated tissue), the characterizing of this biomarker is not much helpful as the tissue must first be isolated from the brain.

2) In all figures, please present single data points.
